# Sex Differences in Associations Between Adolescent Psychopathology and Delinquency

**DOI:** 10.1016/j.jaacop.2024.12.002

**Published:** 2024-12-11

**Authors:** Louise C.S. Smallenburg, Pascalle Spaan, Nina H. Grootendorst-van Mil, Diandra C. Bouter, Witte J.G. Hoogendijk, Maaike Kempes, Sabine J. Roza

**Affiliations:** a Erasmus MC University Medical Center Rotterdam, the Netherlands; b Netherlands Institute for Forensic Psychiatry and Psychology, Utrecht, the Netherlands; c Epidemiological and Social Psychiatric Research Institute (ESPRi), the Netherlands; d Netherlands Institute for Forensic Psychiatry and Psychology, Utrecht, the Netherlands; e Leiden University, Institute of Education and Child Studies, Leiden, the Netherlands

**Keywords:** psychopathology, delinquency, criminal behavior, adolescence, sex differences

## Abstract

**Objective:**

The present study investigated sex differences in the associations between adolescent psychopathology and delinquent behavior over time.

**Method:**

In a Dutch prospective population-based cohort, oversampled on emotional and behavioral problems, we examined the associations between psychopathology, based on multi-informant standardized assessments (mean age, 15 years) and self-reported early delinquency outcomes on average 3 years later in 725 adolescents. Associations between psychopathology (ie, depressive, anxiety, attention-deficit/hyperactivity disorder [ADHD], oppositional defiant and conduct problems, and psychotic experiences) and self-reported early delinquent behavior were tested using logistic regression models. Sex–psychopathology interaction effects were explored.

**Results:**

Of the participating adolescents, 21% reported serious delinquent behavior at follow-up. ADHD and conduct problems at age 15 years were associated with higher odds of serious delinquent behavior at age 18 years (OR = 1.96, 95% CI = 1.21-3.18 and OR = 4.75, 95% CI = 2.32-9.76, respectively). ADHD was associated with serious delinquent behavior in boys, but not in girls. In cross-sectional analyses at age 18 years, having anxiety problems was protectively associated with serious delinquent behavior.

**Conclusion:**

Psychopathological problems are associated with serious delinquent behavior, with variations in the associations observed between adolescent boys and girls. These findings indicate that the pathways linking psychiatric conditions to delinquent behavior differ across sexes.

## Plain language summary

The authors assessed associations between psychopathology and self-reported delinquency in the iBerry Study, a Dutch population-based adolescent cohort at high risk of psychopathology. The study found that attention-deficit/hyperactivity disorder (ADHD) and conduct problems at age 15, over and above substance use and other emotional and behavioral problems, were associated with serious delinquent behavior at age 18. Notably, the association of ADHD with later serious delinquent behavior was present in boys, but not in girls. Having anxiety problems at age 18 was associated with delinquent behavior at the same age. The authors highlight that delinquent behavior may be related to underlying psychopathology with variations across sexes.

Juvenile delinquency is a major problem that exacts a considerable toll, not only on victims and society but also on the development of the adolescents involved in delinquent behavior.^1^ Delinquency peaks in late adolescence.^2^ Although girls end up in detention less often,^3^ the number of female adolescents committing crimes has been increasing.^4^ Although the patterns and rates of delinquent behavior vary from country to country, the causal factors associated with juvenile offending appear to be similar.^5^ Experimental behavior can be considered normative to some extent during the developmental stage of puberty. Nonetheless, committing even a single offense, such as driving under the influence, can have severe consequences. Early identification of boys and girls at high risk for serious delinquent behavior, along with timely intervention, is essential, as juvenile delinquency often leads to multiple negative effects such as continuation of crime, physical and mental health problems, and economic problems in adulthood.^1^ The current study investigated sex differences in associations between psychopathology and delinquency on average 3 years later in 725 adolescents.

Simultaneously with delinquent behavior, psychopathology becomes more apparent during adolescence. The term “psychopathology” describes a broad range of emotional and behavioral problems, including externalizing problems such as attention-deficit/hyperactivity disorder (ADHD), conduct disorder (CD), oppositional defiant disorder (ODD), and internalizing problems such as anxiety and depression, as well as psychotic symptoms.^6^ A meta-analysis showed that the peak age of onset for any psychiatric disorder was 14.5 years^7^, with externalizing psychopathology more prevalent in boys and internalizing disorders more prevalent in girls.^7^^,^^8^

Youth who are involved in delinquent behavior meet criteria for psychiatric disorders more often than youth in the general population.^9^ However, the identification of children at high risk for serious offending remains challenging, and interventions aimed at psychopathology exert inconsistent effects on delinquent behavior.^10^ The association between psychiatric disorders and delinquency is multifactorial, with influences that are sometimes indirect.^11^, ^12^, ^13^, ^14^ First, symptoms of the disorder, such as persecutory delusions in psychosis or poor self-control in ADHD, may in themselves lead to violent or criminal behavior.^11^^,^^14^ Second, a proportion of the association between psychiatric disorders and delinquency may be explained by comorbid substance use.^11^^,^^13^^,^^14^ Third, psychopathology and criminal behavior may share common risk factors or third variables, such as socioeconomic factors, sociodemographic factors, adverse childhood experiences, and/or common underlying genetic predispositions.^11^, ^12^, ^13^, ^14^

Although many previous studies in forensic psychiatry have focused primarily on adults or on associations between disruptive behavioral disorders and youth crime, less attention has been given to adolescents in community-based samples with diverse psychopathology symptoms who engage in delinquent behavior.^15^^,^^16^ A systematic review by Fazel *et al.* on the prevalence of mental disorders (psychotic illness, major depression, ADHD, and conduct disorder) found that approximately 3% of adolescents in correctional facilities had a psychotic disorder, and that adolescents in correctional facilities were about 10 times more likely to suffer from psychosis than their peers in the general adolescent population.^9^ Girls in detention were more often diagnosed with major depression than boys in detention (29% vs 11%). The prevalence of ADHD varied between approximately 12% for boys and 19% for girls. Conduct disorder was the most common of the studied disorders, similarly prevalent across sexes at approximately 53%. In another, cross-sectional, study of 204 incarcerated adolescent boys, 90% were reported to have at least 1 psychiatric disorder: disruptive behavior disorder (75%), psychotic symptoms (34%), ADHD (8%), anxiety disorder (9%), affective disorder (6%), and substance use disorder (55%).^17^ Teplin *et al.* found higher rates of affective disorders among adolescents detained in the state of Illinois, especially among female individuals: more than 20% of female adolescents met diagnostic criteria for a major depressive episode.^18^

Furthermore, externalizing disorders, such as conduct disorder and ADHD, are largely characterized by behavioral symptoms. The behavioral symptoms associated with these externalizing disorders increase the likelihood that adolescents with such symptoms will come to the attention of the juvenile justice system. In our prospective study of adolescents who have not yet come to the attention of the justice system and who self-reported their delinquent behavior, this selection bias is absent, emphasizing the advantage of our research.

Whether associations between psychopathology and delinquency differ depending on sex has not been sufficiently studied so far.^19^ Although some studies have implicitly assumed that risk factors for delinquent behavior in girls are basically the same as those for boys, other studies have identified specific vulnerabilities that affect girls differently.^20^ A review of 30 European studies on risk factors for delinquency in female adolescents, compared with those for adolescent male delinquency, indicated that the risk factors for delinquency that may differ between boys and girls are not rooted in personality traits, such as impulsivity or intellectual disability, or in a risky lifestyle, but rather in individual and family factors. For girls, psychopathology, early maturation, a problematic relationship with their mother and/or teacher, and the level of delinquent behavior among peers appear to be distinct factors.^21^ Regarding psychopathology, girls in justice-involved samples were more likely than boys to report anxiety and affective disorders.^9^^,^^22^ For example, in almost 1,000 randomly selected youth (200 girls) at probation intake in the state of Texas, girls’ rates of anxiety and affective disorders, measured by diagnostic interviews, were higher than those of boys.^22^

In summary, most previous studies have focused primarily on psychiatric disorders within justice-involved samples of boys, and a causal relationship could not be established because of the retrospective or cross-sectional nature of these studies. This underscores the necessity of our prospective study on a wide range of psychopathology in a community-based sample of male and female adolescents.

In this study, we investigated sex differences in associations between psychopathology and delinquent behavior in adolescence. Using data from a large population-based adolescent cohort at high risk for psychopathology, with an equal sex distribution, we used multi-informant standardized assessments of a broad spectrum of emotional and behavioral problems. The sampling method was designed to deliberately include more adolescents at risk for psychopathology, which increases the statistical power of analyses targeting delinquent behavior and mental health outcomes. Because of the relatively large number of female participants compared to those in justice-involved samples, we were able to address whether associations between psychopathology and delinquency differ depending on sex.

Because of the earlier found prevalence rates of internalizing and externalizing problems in juvenile justice–involved samples, we cautiously hypothesized that externalizing problems may be more strongly associated with delinquent behavior in boys, and internalizing problems may be more strongly associated with delinquent behavior in girls.

## Method

### Design, Setting, and Participants

The current study was conducted as part of the iBerry Study, a longitudinal cohort study of 1,022 adolescents at high risk for psychopathology.^23^ The selection of participants started with 16,758 adolescents in the first year of high school (aged 13 years) in the greater Rotterdam area (including rural and urban regions in the Netherlands) who completed the Strengths and Difficulties Questionnaire for youth (SDQ-Y) as part of a standard health screening. The 15% highest-scoring adolescents and a random sample of the 85% lowest-scoring adolescents were included, resulting in a 2.5:1 ratio favoring high-risk adolescents. The study was approved by a Medical Ethical Review Committee. All adolescents and, if applicable, their parents or legal guardians provided written informed consent and completed in-depth psychiatric interviews, questionnaires, and biological measurements. Adolescents received a small monetary compensation.

For the present analysis, data on psychopathology at baseline (T0), conducted between September 2015 and 2019, and self-reported delinquency at first follow-up (T1), conducted between 2019 and 2022, were available for 725 adolescents (70.9%).^24^

### Measures

#### Emotional and Behavioral Problems

Psychopathology was determined based on the multi-informant standardized assessment outcomes of the Achenbach System of Empirically Based Assessment (ASEBA) self-, parent- and teacher-report instruments, respectively; the Youth Self Report (YSR 11-18); the Child Behavior Checklist (CBCL 6-18); and the Teacher’s Report Form (TRF). The *DSM*-oriented scales of the ASEBA were used to measure depressive, anxiety, somatic, ADHD, oppositional defiant, and conduct problems over the past 6 months. The *DSM*-oriented scales have good internal consistency and good test–retest reliability.^25^ Psychopathology was scored (yes/no) using the borderline clinical cut-off, which differs per scale and by sex, and represents scores in the 93th to 100th percentile of the norm group. An adolescent was considered to experience psychopathology if at least 1 of 4 (at T0: adolescent, both parents, teacher), or 1 of 2 (at T1, young adult and 1 of the parents) informants scored above the relevant cut-off for that type of psychopathology.

#### Psychotic Experiences

The 16-item Prodromal Questionnaire—16 (PQ-16) was used to measure psychotic experiences. This self-report questionnaire consists of 16 statements, of which 14 concern positive symptoms and 2 concern negative symptoms with which the adolescent can either agree (1) or disagree (0). Psychotic experiences were scored positive (1) using the cut-off score of 6 or more. The PQ-16 has good concurrent validity and sufficient internal consistency.^26^

#### Delinquent Behavior

Delinquent behavior was measured with an adapted version of the Self-Report Early Delinquency (SRED).^16^^,^^27^ Adolescents were interviewed on how often they engaged in delinquent behavior, such as stealing, vandalism, and violence. In accordance with Van der Laan *et al.*,^16^ we excluded items that did not concern criminal behavior (eg, truancy and substance use). Following Spaan *et al.*, the item “hitting someone at home” was removed because reliability analyses indicated that it did not fit the scale and probably indicates relatively normal sibling fights among youth.^28^ Finally, 23 items with an explicit reference period of 6 months were used at T0.

At T1, the SRED was slightly modified by adding items that were considered better suited for older adolescents, such as selling drugs, fraud, driving under the influence, sexual assault, animal cruelty, and cybercrime. At T1, young adults reported on 35 items referencing a period of 2 years prior. We categorized items into different types of delinquent behavior, based on the Statistics Netherlands standard crime classification.^29^

Crimes were considered light or serious based on their potential sanction severity in Dutch criminal law. Serious crimes weighed more heavily than light crimes when calculating the total score. A light crime was scored as follows: not committed (0), committed 1 to 3 times (1), or committed 4 or more times (2). A serious crime was scored as follows: not committed (0), committed 1 time (4), or committed 2 or more times (8). Scores were summed, with higher scores indicating more serious and/or more frequent delinquent behavior. Delinquency scores were dichotomized based on the top 10%, in accordance with Van der Laan *et al.,* resulting in a cut-off score of 8 or more. We used the same cut-off scores at T1 and T0. In our sample of 725 adolescents who completed the SRED at T1, internal consistency was good (α = 0.85).

#### Other Measurements

Adolescents and accompanying parents completed questionnaires on sex assigned at birth (male or female), age, country of birth, education level, and household income. Ethnic origin was categorized as 1 of the parents being born abroad. Intelligence score (T0) was estimated using 2 subtests of a Dutch non-verbal IQ test: Snijders–Oomen Non-verbal Intelligence Test—Revised (SON-R 6-40). The SON-R 6-40 is relatively insensitive to cultural differences.^30^ The subtests’ analogies and categories were conducted; both measure general reasoning skills. These subtest scores correlate strongly with total intelligence scores (respectively, 0.68 and 0.59). Substance use was measured at T1 using a self-constructed questionnaire, and dichotomized into frequent alcohol use (lifetime use of alcoholic beverages 40 times or more) or not, as well as into frequent drug use (lifetime use of any drug 3 times or more) or not.

### Data Analyses

Differences in baseline characteristics between participating boys and girls were compared with independent *t* tests (continuous variables), χ^2^ tests (categorical variables), and Mann–Whitney *U* tests (non–normally distributed variables). To examine whether attrition was selective, we compared several baseline characteristics of adolescents with T1 delinquency data (responders, n = 725) to those with missing T1 delinquency data (non-responders, n = 297).

We used logistic regression models to study the association between psychopathology (binary variables) at T0 and delinquent behavior at T1 (binary outcome). A hierarchical approach was used to add predictors to our models with delinquency as the dependent variable. In the first step, we jointly introduced psychopathology problems as independent variables and adjusted for a set of demographic confounders, namely, age, sex, ethnic origin, intelligence, household income, and substance use. As a second step, we examined whether potential interaction effects were of added value to the first model, by adding all sex–psychopathology interaction terms into the model and evaluating whether there were improvements in model fit as indicated by the χ^2^ tests and the Nagelkerke *R*^*2*^ values.^12^ Sex–psychopathology interaction terms were visualized to facilitate interpretation of the findings. To examine whether the associations between psychopathology and delinquent behavior were time specific, we performed similar analyses to study the cross-sectional associations between psychopathology and delinquency at T1 (Table S1, available online). In secondary analyses, we stratified analyses based on adolescents’ sex, to test the robustness of the findings in these separate groups with more parsimonious models that do not require inclusion of the sex–psychopathology interaction terms. Thus, a total of 4 logistic regression models were conducted.

We used SPSS Statistics Version 28 for all analyses.^31^ A *p* value of less than .05 was considered statistically significant, except for studying the potential interaction effect of sex. To ensure that potential sex differences were not missed because of insufficient power, we chose to raise the type I error rate to 10% for potential modification of associations by sex.^32^

#### Multiple Imputation

We included only those adolescents who provided data on psychopathology at T0 or T1 and on the primary outcome, serious delinquent behavior at T1. We used multiple imputation to correct for missingness of covariate information. In some adolescents, data were missing for demographic characteristics (0.0%-11.6%), alcohol use (3.2%), drug use (2.1%), and serious delinquent behavior at T0 (2.3%) because of declined interviews or unreturned questionnaires. Of the 118 adolescents with missing covariate data (on estimated IQ, ethnic background, household income, drug use, or alcohol use), the majority were missing data on only 1 covariate (n = 52) or 2 covariates (n = 60), and only 6 adolescents missed data on 3 or 4 covariates. None of the adolescents missed all covariate data. We assumed that these values were missing at random; that is, we assume that missingness was related to variables on which we did have information. For example, adolescents with higher SDQ screening scores may have more problems that hinder them from participating in follow-up measurements. In addition to all variables that were a part of our main analyses (ie, delinquency, sex, psychopathology, sex–psychopathology interactions, age, ethnic origin, estimated IQ, household income, drug and alcohol use), we added auxiliary variables that likely informed missingness. Auxiliary variables theoretically assumed to be related to missingness and used for imputation were adolescents’ SDQ screening status, education level at T0, smoking, alcohol and drug use at T0, and age at T1. We used multiple imputation to replace these covariate missing values with 5 imputed datasets. Scale variables were imputed based on predictive mean matching, and binary variables based on logistic regression. Regression coefficients were pooled across imputation sets automatically, based on the Rubin rules, which take into account both within and between imputation variance.^33^ For χ^2^ tests and Nagelkerke *R*^*2*^*,* which were not pooled automatically, median values were reported.^34^

## Results

### Sample Characteristics

Characteristics of the study subjects by sex are presented in Table 1. A total of 388 participants (53.5%) were female. The mean age at T0 was 14.9 years and at T1 was 17.9 years. The majority of adolescents (82.5%) reported alcohol use 40 times or more lifetime, although minors (<18 years of age) are prohibited to buy alcoholic beverages under Dutch law. Female adolescents reported less drug use than male adolescents (31.4% vs 43.2%). In total, 21% of the 725 adolescents in our sample were classified as serious delinquent at T1. Girls reported less serious delinquent behavior compared to boys, both at baseline (5.2% vs 14.2%) and at T1 (10.3% vs 33.2%).

**Table 1:** Characteristics of the Study Population

Adolescent	Total n = 725	Girls n = 388, 53.5%	Boys n = 337, 46.5 %	*p*
Age at T0, y, mean (± SD)	14.9 (± 0.9)	14.8 (± 0.9)	14.9 (± 0.9)	.169
Age at T1 y, mean (± SD)	17.9 (± 0.7)	17.9 (± 0.7)	18.0 (±0.8)	.314
Age of accompanying parent at birth of adolescent, y, mean (± SD)	31.9 (± 5.5)	31.7 (± 5.7)	32.0 (± 5.3)	.437
Western ethnic background, n (%)	572 (85.4)	308 (86)	264 (84.6)	.604
Estimated IQ^a^, mean (± SD)	99.3 (± 13.6)	99.4 (± 13.1)	99.1 (±14.1)	.876
Education level T0, n (%)				.387
Pre-vocational secondary	305 (43.9)	163 (44.2)	142 (43.6)	-
Higher general secondary	165 (23.7)	93 (25.2)	72 (22.1)	-
Pre-university	165 (23.7)	87 (23.6)	78 (23.9)	-
Combined	60 (8.6)	26 (7)	34 (10.4)	-
Net monthly household income <2000 euro, n (%)	98 (15.3)	59 (17.3)	39 (13.0)	.131
Alcohol use T1 >40 times, n (%)	579 (82.5)	313 (82.8)	266 (82.1)	.806
Drug use T1 ≥3 times, n (%)	262 (36.9)	119 (31.4)	143 (43.2)	**.001**
Serious delinquent behavior T0, n (%)	68 (9.6)	20 (5.2)	48 (14.2)	**<.001**
Serious delinquent behavior T1, n (%)	152 (21)	40 (10.3)	112 (33.2)	**<.001**
Psychopathology^b^, n (%)				
Depressive problems	222 (30.9)	141 (36.8)	81 (24.1)	**<.001**
Anxiety problems	186 (25.9)	113 (29.5)	73 (21.7)	**.017**
Somatic problems	197 (27.5)	111 (29.1)	86 (25.7)	.311
Attention deficit/hyperactivity problems	273 (38)	153 (39.9)	120 (35.7)	.243
Oppositional defiant problems	104 (14.5)	67 (17.5)	37 (11)	**.014**
Conduct problems	85 (11.8)	55 (14.4)	30 (8.9)	**.024**
Psychotic experiences T0, n (%)	154 (22)	90 (24.3)	64 (19.5)	.126

Note: Results based on non-imputed data.

a Snijders–Oomen Non-verbal Intelligence Test—Revised corrected for Flynn effect.

b One or more informants (adolescent, parent[s) and/or teacher) above borderline cut-off score of 93rd percentile Youth Self Report, Child Behavior Checklist, and/or Teacher’s Report Form. Sixteen-item Prodromal Questionnaire—16 cut-off score ≥6.

### Response Analyses

Analyses of missing data revealed that non-responders at T1 (n = 297) were more often male (54.9% vs 46.5%, χ^2^[1] = 5.95, *p* = .015), older at baseline (mean difference 0.47 years, 95% CI = 0.34-0.60, t[496.681] = 7.18, *p* < .001), with lower levels of education (7.9% higher education vs 23.7%, χ^2^(3) = 38.24, *p* < .001), and lower household incomes (32.3% < €2000 vs 15.3%, χ^2^[1] = 31.13, *p* < .001) than responders. Furthermore, non-responders were more often categorized with serious delinquent behavior at T0 (14.8% vs 9.6%, χ^2^[1] = 5.27, *p* = .022), more often reported frequent alcohol use (93.4% vs 82.5%, χ^2^ = 5.97, *p* = .015), and were more likely to have ADHD problems (38% vs 29.8%, χ^2^[1] = 5.90, *p* = .015) and anxiety problems (25.9% vs 16.8%, χ^2^[1] = 9.31, *p* = 0.002), compared to responders.

### Prevalence of Delinquent Behavior

Table 2 shows delinquent behavior item prevalences at T0 and T1. Most reported was theft of services (eg, going to the cinema or traveling by train without paying). Approximately 10% of the adolescents reported traffic offenses, possession of large quantities of drugs (more than the user amount) or carrying a weapon, even though possession of weapons is prohibited in the Netherlands. In all, 83.1% of the participating adolescents at T0 reported having no prior contact with the police; however, official records with information about prior involvement with the criminal justice system were not available.

**Table 2:** Items and Prevalence by Delinquency Type, Categorized as Light or Serious Crimes

Delinquent behavior	Light (L) or serious (S)	Prevalence T0 (1+ times, %)	Prevalence T1 (1+ times, %)
Violence			
Joining a fight	L	14,6	15,6
Hitting someone in public	S	14,0	11,2
Hitting someone, resulting in a need for medical care	S	2,2	5,7
Carrying a weapon	L	11,5	10,5
Using a weapon	S	0,3	2,2
Theft by force	S	0,4	0,8
Stalking	L	—	2,9
Threatening someone face-to-face	S	—	7,5
Sexual assault	S	—	0,9
Animal cruelty	S	—	1,4
Property crimes			
Theft at home	L	7,7	4,4
Theft, value < €10	L	17,0	18,7
Theft, value €10 - €100	S	2.2	4,3
Theft, value > €100	S	1.7	2,3
Fencing	S	1,7	4,0
Attempted burglary	S	2.0	8,0
Theft of services	L	—	23,7
Government fraud	L	—	4,8
Fraud/deceit	L	—	4,4
Cybercrime			
Threatening someone by phone or social media	S	—	6,2
Hacking	L	—	2,3
Privacy invasion	L	—	3,9
Non-consensual sharing of intimate images	S	—	2,5
Vandalism or public order			
Vandalizing family property	L	7,6	9,4
Vandalizing public property	L	8,5	12,8
Graffitiing	L	9,4	8,0
Gang participation	S	1.2	1,8
Setting fires	L	11,8	13,2
Name-calling	L	—	4,1
Drug crimes			
Dealing soft drugs	S	—	7,7
Dealing hard drugs	S	—	2,6
Drugs possession	L	—	11,4
Traffic offenses			
Joyriding	L	—	9,9
Driving under the influence	L	—	11,4
Hit-and-run	L	—	0,4

Note: Obtained via Self-Report Early Delinquency (SRED).

### Psychopathology and Delinquency

Of the participants, 30.9% reported or were reported with depressive problems, 25.9% with anxiety problems, 38% with ADHD problems, 14.5% with oppositional defiant problems, and 11.8% with conduct problems (Table 1). In total, 22% of the participants reported psychotic experiences above the cut-off score at T0. Girls more often had depressive, anxiety, oppositional defiant, and conduct problems as compared to boys. On the other hand, boys were more often categorized as serious delinquents, both at T0 and at T1. Psychotic experiences did not differ between girls and boys, nor between serious and non-delinquency or minor delinquency.

Table 3 summarizes the main results. We introduced all types of psychopathology at age 15 years together in 1 model and adjusted the model for other important determinants of delinquency, including substance use. In the fully adjusted model, having ADHD problems and conduct problems at age 15 was associated with a higher odds of serious delinquent behavior at age 18. We found no indication that internalizing problems, oppositional defiant problems, or psychotic experiences at baseline were associated with serious delinquent behavior at follow-up, over and above the effects of ADHD and conduct problems.

**Table 3:** Longitudinal Associations of Psychopathology at Baseline on Delinquent Behavior at Follow-up

Model 1: Psychopathology at age 15 y^a^^,^^b^	Serious delinquent behavior at age 18 y		
	OR	95% CI	*p*
Depressive problems	0.74	0.41-1.32	.307
Anxiety problems	0.80	0.43-1.48	.468
Somatic problems	0.76	0.45-1.30	.323
ADHD problems	**1.96**	1.21-3.18	.006
Oppositional defiant problems	0.92	0.46-1.83	.801
Conduct problems	**4.75**	2.32-9.76	< .001
Psychotic experiences	1.31	0.74-2.32	.358
**Model 2: including interaction terms by sex**^c^^**,**^^d^			
Depressive problems	0.66	0.31-1.42	.289
Anxiety problems	0.50	0.22-1.21	.092
Somatic problems	0.79	0.41-1.55	.496
ADHD problems	**1.93**	1.07-3.47	.028
Oppositional defiant problems	0.93	0.37-2.39	.885
Conduct problems	**8.92**	2.73-25.21	<.001
Psychotic experiences	1.23	0.59-2.59	.578
Sex × depressive problems	1.12	0.32-3.85	.863
Sex × anxiety problems	3.44	0.95-12.41	.060
Sex × somatic problems	0.85	0.27-2.68	.784
Sex × ADHD problems	1.16	0.40-3.38	.787
Sex × oppositional defiant problems	0.94	0.23-3.89	.930
Sex × conduct problems	0.26	0.05-1.30	.173
Sex × psychotic experiences	1.05	0.32-3.43	.930

Note: Significant odds ratios are shown in boldface type. ADHD = attention-deficit/hyperactivity disorder; OR = odds ratio; *R*^*2*^ = Nagelkerke *R*^*2*^.

a Results based on imputed data. All *DSM*-oriented scales and 16-item Prodromal Questionnaire—16 (PQ-16) scores are introduced jointly. The model is adjusted for adolescents’ age, sex, ethnic origin, estimated IQ, household income, and drug and alcohol use.

b χ^2^ = 207.57 (*p* <.001)*; R*^*2*^ = 0.41.

c Results based on imputed data. All *DSM*-oriented scales and PQ-16 scores are introduced jointly, together with their interaction terms by sex. The model is adjusted for age, sex, ethnic origin, estimated IQ, household income, and drug and alcohol use.

d χ^2^ = 214.07 (*p* <.001)*; R*^*2*^ = 0.41.

Table S1 shows the cross-sectional associations between psychopathology and delinquent behavior at age 18 years. Also cross-sectionally, ADHD and conduct problems were independently associated with a higher odds of serious delinquent behavior. Moreover, having anxiety problems was associated with a lower odds of serious delinquent behavior (OR = 0.23, 95% CI = 0.10-0.54, *p* <.001).

### Sex Differences in Psychopathology and Delinquency

**Figure 1:**
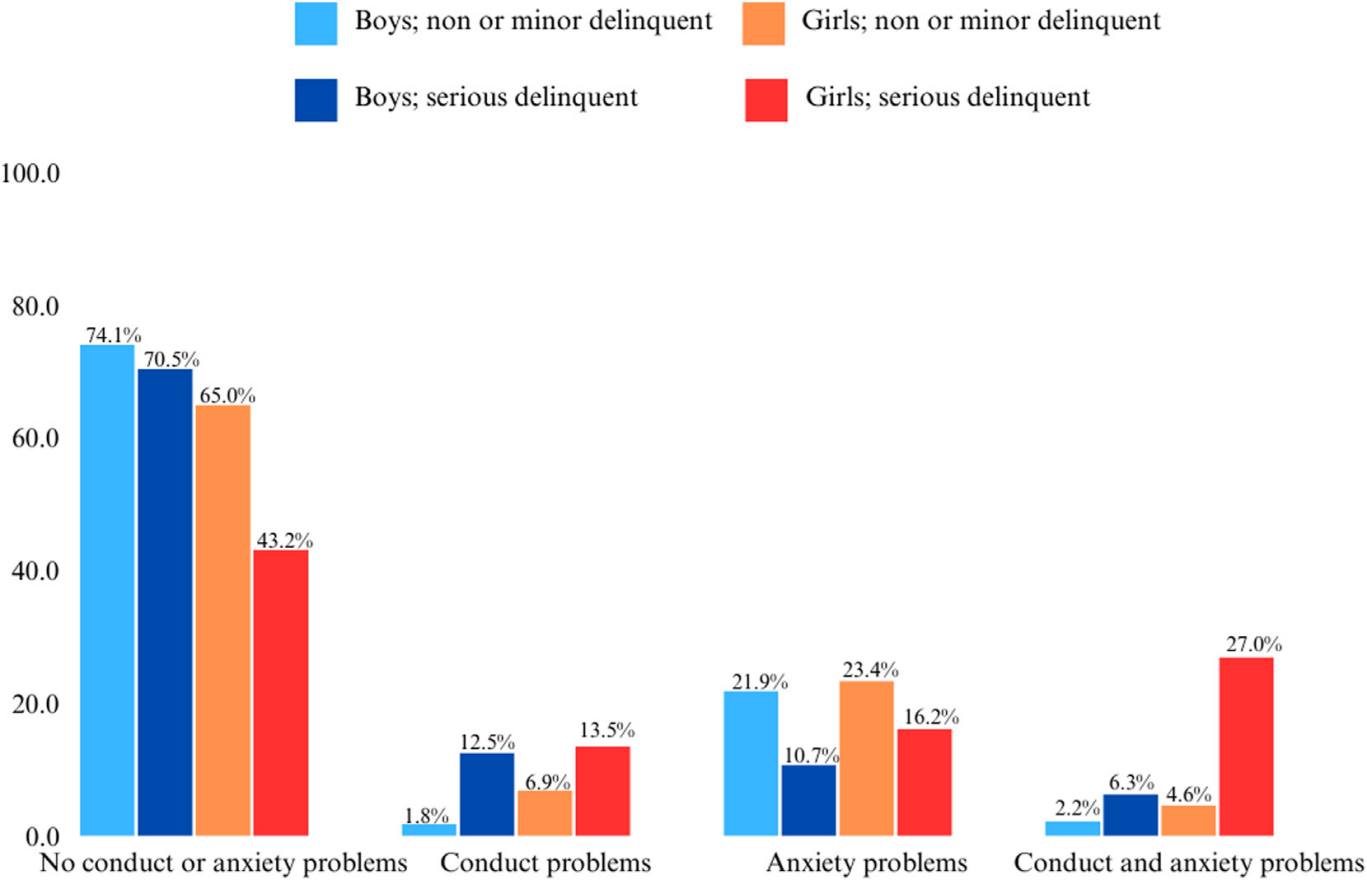
Percentage of Adolescent Boys and Girls with Conduct and/or Anxiety Problems at Baseline by Delinquency at Follow-Up

To study sex-specific effects, we introduced interaction terms of psychopathology by sex in our main model. We observed a potential interaction effect between sex and anxiety problems at age 15 years on serious delinquent behavior at age 18 (*p* = .06). We visualized potential sex-specific effects in Figure 1, which indicates that comorbidity of anxiety and conduct problems at age 15 tends to predict more serious delinquent behavior at age 18 among girls, but not among boys.

As secondary analyses, we performed stratified analysis post hoc in adolescent boys and girls separately. In the fully adjusted model, ADHD and conduct problems at age 15 years were associated with a higher odds of serious delinquent behavior at age 18 in boys (respectively, OR = 1.97, 95% CI = 1.10-3.52, *p* = .02 and OR = 7.13, 95% CI = 2.38-21.4, *p* < .001). In girls, only conduct problems at age 15 years were independently associated with serious delinquent behavior at age 18 (OR = 2.93, 95% CI = 1.00-8.61, *p* = .05). In boys, anxiety problems at age 15 years seem to be protective for serious delinquent behavior (OR = 0.49, 95% CI = 0.22-1.08, *p* = 0.08), which we did not observe in girls (OR = 1.56, 95% CI = 0.52-4.65, *p* = 0.43).

### Sensitivity Analysis

We ran all of the logistic regression models 1 more time while correcting for continuous delinquency scores at T0, to estimate whether changes in delinquent behavior from T0 to T1 were similarly associated with psychopathology. We did not do this for our main analyses, as it is part of the expected pathway from psychopathology at baseline to delinquency at T1. We found that all results were in the same direction and had similar effect sizes while controlling for baseline delinquency. For the sex-stratified models, we observed a slight change. Among girls, only ADHD problems at age 15 years (OR = 2.80, 95% CI = 1.06-7.43, *p* = .038), rather than conduct problems (OR = 2.74, 95% CI = 0.89-8.51, *p* = .081), were associated with any change in delinquent behavior from T0 to T1, that is, the onset of new serious delinquent behavior.

## Discussion

In the current study in a population-based adolescent cohort at high risk for psychopathology, we found that 21% of participating adolescents reported serious delinquent behavior at follow-up, whereas this percentage was approximately 10% at baseline. We showed that ADHD and conduct problems at age 15 years, over and above emotional and other behavioral problems as well as substance use, were associated with serious delinquent behavior at age 18. Notably, the association of ADHD with later serious delinquent behavior was present in boys, but not in girls. We found no evidence for an association between psychotic experiences and delinquency in either sex. Albeit not statistically significant at a 5% level, anxiety problems at age 15 years tended to be protective against serious delinquent behavior at age 18 in boys, but not in girls. Furthermore, having anxiety problems at age 18 was protectively associated with delinquency outcome at the same age.

The increase in the percentage of adolescents reporting serious delinquent behavior from 10% at baseline to 21% at follow-up can only be partially explained by the inclusion of more items in the self-reported early delinquency questionnaire at follow-up, as several of the newly added items, such as sexual assault, are rarely reported. Considering the age–crime curve,^2^ we also expected an increase in the number of adolescents reporting serious delinquent behavior.

As expected, due to partial symptom overlap between conduct problems and our measure of delinquent behavior, we found that conduct problems at age 15 years were associated with delinquent behavior at age 18 years. In addition to conduct problems, ADHD is also a well-known risk factor for delinquency in early adolescence.^9^^,^^11^ Our study adds to previous research, as it encompasses not only ADHD, ODD, and conduct problems, but also a broader range of psychopathology, including anxiety and depressive symptoms, and psychotic experiences. Moreover, in contrast to results in adolescents already involved in the juvenile justice system, we found no indication that ADHD problems in girls are an independent risk factor for delinquency. That is, in our analyses, conduct problems at age 15 years in girls were related to later delinquency, and having ADHD did not add significantly to this prediction. Fazel *et al.* described an even higher prevalence of ADHD among incarcerated adolescent girls (18.5%) than among boys (11.7%).^9^ Other studies, however, found that female individuals with ADHD were reported to have less delinquent, aggressive, or rule-breaking behavior, but more depressive symptoms than male individuals.^35^^,^^36^ Taken together, more well-powered longitudinal studies involving both sexes are needed to draw conclusive results regarding sex differences in the associations between ADHD and CD, and delinquent behavior. In this regard, attention should also be given to trauma-related disorders as a well-researched risk factor for delinquent behavior,^37^ considering the potential comorbidity and overlap between disorders such as ADHD and trauma-related disorders.

For anxiety problems, our findings suggest that boys were less likely than those without such problems to commit serious delinquent behavior, whereas in girls the specific comorbidity of conduct and anxiety problems seem to have the largest effect on later delinquency outcomes. Our findings add to prior research, discussed in the introductory section of this article,^20^, ^21^, ^22^ showing that internalizing problems were more prominent in female than in male adolescents committing delinquency. However, these studies have been conducted in preselected, justice-involved samples, based on outcome, making causality difficult to establish. Prior longitudinal studies point to contradicting evidence regarding the direction and potential causal pathway linking anxiety to delinquent behavior. A longitudinal survey in 503 boys of the Pittsburgh Youth Study indicated that depression and anxiety were consequences of offending rather than causes.^38^ Our finding that anxiety problems were cross-sectionally associated with a lower odds of serious delinquent behavior is in line with a longitudinal study of approximately 1,450 Greek Cypriot male adolescents, which showed that anxiety negatively predicted delinquency.^39^ On the other hand, there have been researchers who showed that anxiety was linked to later delinquent behavior, as indicated by the systematic review of Fazel *et al.* In sum, knowledge regarding the impact of anxiety on delinquent behavior derives primarily from samples of boys or men. Our study contributes to prior work, as longitudinal research among samples of girls who have not yet come into contact with the justice system is scarce. It is important to note, regarding sex differences in psychopathology, that studies in youth involved in delinquent behavior often lack an adequate number of girls, making them frequently underpowered. Even though increased attention has arisen to sex-specific aspects in psychiatry in recent years, little is known about the mechanisms that underlie sex differences in psychopathology.^40^^,^^41^

Studying a potential 3-way-interaction of sex, anxiety, and conduct problems would need a much larger study sample. However, within our sample we saw that 18-year-old girls who reported serious delinquent behavior had more often combined anxiety and conduct problems at age 15. This finding is in line with clinical findings in 211 Dutch adolescent detained girls sentenced under criminal or civil law, who often have externalizing disorders such as CD and ODD and problems with substance use, but also internalizing disorders or other comorbid disorders.^42^ Another Dutch study found a link between childhood internalizing problems and serious delinquent behavior in adolescent girls involved in the justice system, partly determined by simultaneous externalizing problems.^43^ Although some earlier studies reported that internalizing problems increase the risk of serious offending, especially among girls,^9^^,^^20^ our study confirms and elaborates these findings in girls who are not yet involved in the juvenile justice system. Furthermore, previous research by our research group^44^ found that dual-harm, defined as concurrent aggression toward oneself and others, frequently occurs both in boys and in girls. The combination of internalizing and externalizing problems in girls should increase awareness. Regarding early identification and timely interventions, professionals in child and adolescent psychiatry and youth care should especially be aware of (underlying) anxiety problems in girls who present with conduct problems.

In our sample, girls at age 15 years experienced not only more internalizing problems than boys, but also more externalizing problems. These somewhat counterintuitive observation may illustrate sex differences in pubertal development at this specific age,^45^ but might also reflect time trends. As the number of female suspects of crime among minors has increased over time,^4^ cut-off values of the *DSM*-oriented scales for girls regarding oppositional defiant and conduct problems might have been outdated. Girls may have shifted toward the values of boys across recent years, and sex-specific cut-offs for the *DSM*-oriented scales of ASEBA may no longer be appropriate. Whereas the conduct problems subscale shows overlapping items with the self-reported offending items of the SRED at T1, the oppositional defiant problems subscale is not about criminal offenses, but consists of items such as disobedience at home or school and temper. It is tempting to speculate that the oppositional defiant and conduct problems scales are influenced by broader increases in behavioral problems in girls than just delinquency. Furthermore, girls do seem to have smaller chances of being suspected of crime than boys, which may be explained by their involvement in more covert offense, their playing a more social role in group criminality, or perceptions within the police force. It appears that the police force is more likely to regard girls as victims rather than as offenders, and to perceive boys as less communicative than girls.^46^ As a third potential explanation, the higher percentage of girls with oppositional defiant and conduct problems in our study sample may be explained by reporter bias. Parents or teachers may be more inclined to label girls’ behavior as problematic compared to that of boys, for instance, because oppositional defiant and conduct problems in girls are often more covert than the more overt externalizing behaviors seen in boys.^46^

In adults, most research on the association between psychopathology and delinquency reported on the increased risk of violence due to psychotic disorders. In addition to other factors such as drug use being explanatory for offending behavior in adults, these studies showed a small but significant association between psychotic disorders and (mainly violent) offending.^14^ In our sample, however, we studied psychotic experiences rather than psychotic disorders. Our sample is relatively young, and whether any of the study participants will develop psychotic disorders in the future is still unclear. However, it is important to note that even among those with significant psychotic experiences, the majority are unlikely to develop psychotic disorders.^47^ Furthermore, on a group level, the joint effects of ADHD and conduct problems are probably (much) higher than any effect of psychotic experiences in the prediction of later delinquency.^48^

This study in a large group of adolescents has several strengths, including the relatively high proportion of girls, its longitudinal design with multi-informant standardized assessment outcomes of a wide range of psychopathology, and multiple methods of data assessment. However, some limitations should be noted. First, selection effects may have influenced our findings. Our response analyses indicate that there was a selective dropout of adolescents with known risk factors for future delinquency. Dropouts more often reported serious delinquent behavior, alcohol use, and ADHD at age 15 years. Selective attrition might have introduced selection bias, which is not necessarily an underestimation of associations between psychopathology and delinquent behavior. Second, although we expect that our results from a study sample in a medium-sized area in the Netherlands, which includes both rural and urban regions, are generalizable to other countries and cultures,^5^ replication of the findings in other settings, such as more diverse urban areas with higher rates of juvenile violence, is needed to confirm this hypothesis. Third, the use of self-reported early delinquency data is inherently different from that of official records.^49^ Although we believe that adolescent self-report on delinquency is probably more sensitive than parent or teacher report, the risk of socially desirable reporting is noteworthy. Official records have their limitations as well, because they measure only detected and reported delinquency. Fourth, although we accounted for several possible confounding factors, we cannot rule out residual confounding, as there are multiple confounders that could moderate the link between psychopathology and involvement in delinquent behavior, such as a low socioeconomic status and exposure to violence, drugs, and peers involved in delinquent behavior.^50^ Although we did not consider baseline delinquent behavior as a confounder in the association between psychopathology at baseline and delinquent behavior at follow-up, our results reflect associations, and we cannot be certain of whether they indicate causal effects.

In conclusion, our longitudinal results in a large population at high risk for psychopathology provide further evidence that boys and girls may differ in psychopathology related to delinquent behavior. Our findings highlight that delinquent behavior may be a potential psychopathology-related negative outcome in adolescents. The association between psychopathology and delinquent behavior among girls is particularly important, not only because girls are nowadays more often arrested for crime-related behavior than in the past,^4^ but also because intergenerational transmission of delinquent behavior may be stronger from mother to offspring than from father to offspring.^51^ Future studies should address potential underlying causal factors of sex differences in the associations between psychopathology and delinquent behavior, such as peer relationships, puberty timing, impulsivity, or executive functioning.

## CRediT authorship contribution statement

**Louise C.S. Smallenburg:** Writing – review & editing, Writing – original draft, Visualization, Methodology, Investigation, Funding acquisition, Formal analysis, Data curation, Conceptualization. **Pascalle Spaan:** Writing – review & editing, Writing – original draft, Visualization, Investigation, Formal analysis, Data curation. **Nina H. Grootendorst-van Mil:** Writing – review & editing, Project administration, Funding acquisition. **Diandra C. Bouter:** Writing – review & editing, Data curation. **Witte J.G. Hoogendijk:** Writing – review & editing, Project administration. **Maaike Kempes:** Writing – review & editing, Writing – original draft, Supervision, Project administration, Methodology, Funding acquisition, Conceptualization. **Sabine J. Roza:** Writing – review & editing, Writing – original draft, Supervision, Project administration, Methodology, Funding acquisition, Data curation, Conceptualization.
