# Supplementary material for: Sex Differences in Associations Between Adolescent Psychopathology and Delinquency
Source: JAACAP Open. 2024 Dec 11;3(4):972–83. doi: 10.1016/j.jaacop.2024.12.002 (PMC12684462; doi:10.1016/j.jaacop.2024.12.002)
Supplement: Supplemental Table S1 [file mmc1.docx]

| **Table S1** *Cross-sectional Associations of Psychopathology on Delinquency at follow-up* | | | |  |
| --- | --- | --- | --- | --- |
|  | **Serious Delinquent Behavior at age 18** | | | |
| **Model 1: Psychopathology at age 18^a*^** | **OR** | **95% CI** | ***p*** | |
| Depressive problems | 0.98 | (0.56-1.73) | .950 | |
| Anxiety problems | **0.23** | (0.10-0.54) | <.001 | |
| Somatic problems | 1.17 | (0.65-2.11) | .610 | |
| ADHD problems | **1.80** | (1.07-3.03) | .026 | |
| Oppositional defiant problems | 1.98 | (0.81-4.83) | .132 | |
| Conduct problems | **4.78** | (2.12-10.81) | <.001 | |
| Psychotic experiences | 1.70 | (0.83-3.47) | .147 | |
| **Model 2: including interaction terms by sex^b**^** |  |  |  | |
| Depressive problems | 1.06 | (0.52-2.17) | .865 | |
| Anxiety problems | **0.23** | (0.09-0.55) | <.001 | |
| Somatic problems | 1.04 | (0.47-2.30) | .933 | |
| ADHD problems | 1.65 | (0.86-3.18) | .133 | |
| Oppositional defiant problems | 2.81 | (0.51-15.60) | .239 | |
| Conduct problems | **6.26** | (1.72-22.86) | .005 | |
| Psychotic experiences | 1.77 | (0.66-4.70) | .256 | |
| Sex * depressive problems | 0.69 | (0.21-2.26) | .540 | |
| Sex * anxiety problems | 1.73 | (0.67-4.48) | .259 | |
| Sex * somatic problems | 1.19 | (0.37-4.28) | .717 | |
| Sex * ADHD problems | 0.98 | (0.41-3.45) | .756 | |
| Sex * oppositional defiant problems | 0.72 | (0.09-5.80) | .755 | |
| Sex * conduct problems | 0.57 | (0.10-3.23) | .526 | |
| Sex * psychotic experiences | 0.91 | (0.22-3.69) | .894 | |
| *Note.* Significant odds ratios are in bold.  Abbreviations: CI, Confidence Interval; OR, Odds Ratio; *R^2^*, Nagelkerke's *R^2^*.  ^a^ Values are odds ratios (95% confidence intervals). All DSM-oriented scales and PQ-16 scores are introduced jointly. The model is adjusted for adolescents’ age, sex, ethnic origin, estimated IQ, household income, drug and alcohol use.  ^*^ *χ^2^* 228.47 *(p* <.001)*;* R*^2^* 0.43  ^b^ Values are odds ratios (95% confidence intervals). All DSM-oriented scales and PQ-16 scores are introduced jointly, together with their interaction terms by sex. The model is adjusted for age, sex, ethnic origin, estimated IQ, household income, drug and alcohol use.  ^**^ *χ^2^* 232.41 *(p* <.001)*;* R*^2^* 0.44 | | | |  |
